# Photos and rendered images of LEGO bricks

**DOI:** 10.1038/s41597-023-02682-2

**Published:** 2023-11-18

**Authors:** Tomasz Maria Boiński

**Affiliations:** Gdańsk University of Technology, Faculty of Electronics, Telecommunications and Informatics, Gdańsk, 80-233 Poland

**Keywords:** Computer science, Scientific data, Information technology

## Abstract

The paper describes a collection of datasets containing both LEGO brick renders and real photos. The datasets contain around 155,000 photos and nearly 1,500,000 renders. The renders aim to simulate real-life photos of LEGO bricks allowing faster creation of extensive datasets. The datasets are publicly available via the Gdansk University of Technology “Most Wiedzy” institutional repository. The source files of all tools used during the creation of the dataset were made publicly available via GitHub repositories. The images, both photos and the renders were annotated with the unique brick ID and category from the official LEGO catalog. The proposed datasets are stored in easy-to-read formats and are labeled via directory structure allowing easy manipulation and conversion of metadata to other formats.

## Background & Summary

LEGO bricks, thanks to the availability of a vast array of shapes and colors can be used to build virtually any, both very simple and very complex, constructions. The process, however, to be enjoyable requires that the bricks are properly sorted and arranged. Without that, the building process consists mainly of searching for proper bricks in a big pile of LEGO, which is highly discouraging. The same can be said concerning other activities involving a large number of usually small elements, like construction, collection arrangement, etc.

In the case of LEGO bricks, sorting can be done by both color and shape. Sorting bricks by color only is not very efficient as different shapes tend to blend and are difficult to distinguish. On the other hand, the differently colored bricks can be easily picked from the pile of similarly shaped ones^1^. Still, with over 3700 different LEGO parts^2^ (and the number is constantly growing) even disregarding the color makes the problem of LEGO brick sorting quite complex and time-consuming, even despite the attempts made to optimize the sorting process (e.g.^3^).

The proposed datasets started as part of a solution to such a problem. The author’s collections contains over 50,000 bricks spanning across multiple boxes. Browsing through such an amount of bricks is greatly discouraging. In our research, we aimed at the creation of an AI-powered LEGO sorting machine, as there are no commercially available solutions, and those created by fans are either limited or do not show the building details^4,5^.

**Fig. 1:**
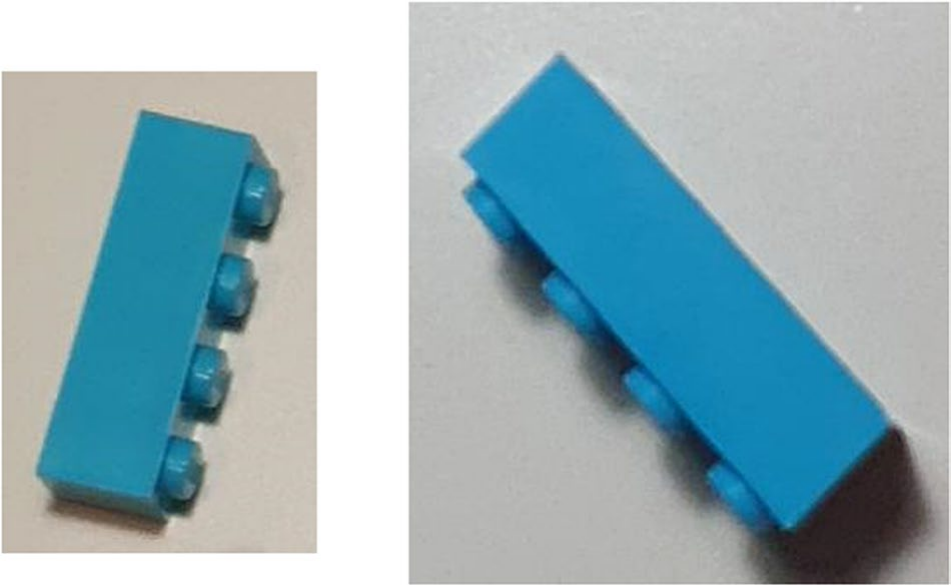
Difficult to distinguish cases for LEGO bricks 3010 (left) and 3001 (right).

To train neural networks a lot of data is needed. Unfortunately, there is no public LEGO bricks dataset available. The LEGO collectors sites like Rebrickable^6^ contain only a limited set of images for each brick, usually viewed from a single angle only (usually 45° top-down view). There is, however, a database of 3D models of LEGO bricks in the form of the LDraw library^7^. Using it requires however extensive computing power to render life-like images. The gathering of real-life LEGO photos requires manual sorting of bricks and manual labeling of gathered images, which, considering the number of brick shapes and colors, would be very time-consuming. By publishing our proposed datasets, we aimed to eliminate this step for other researchers who might be interested in the creation of similar sorting solutions. The images from the datasets could be also used for the publication of fan web pages or as a use case and benchmark for verifying model qualities against in some cases very hard-to-distinguish cases, e.g. for bricks 3001 and 3010 lying on a side as seen in Fig. 1.

Our work was focused on distinguishing bricks by shape. As such the proposed datasets are shape oriented. Both the renders and real photos contain bricks in random colors so that the neural network can be trained to disregard the color or decals on the bricks.

The series consists of 5 datasets:

- LDRAW-based renders of LEGO bricks moving on a conveyor belt with extracted models^8^,
- Tagged images with LEGO bricks^9^,
- Tagged images with LEGO bricks part 2^10^,
- Images of LEGO bricks^11^,
- LEGO bricks for training classification network^12^.

The purpose of this dataset series is to provide researchers with life-like images of LEGO bricks allowing work on multi-class object recognition, e.g. an AI-based sorting machine, without a need to invest the time and effort into the creation of required datasets.

## Methods

The dataset series consists of renders and real photos. Both types of images were obtained differently.

### LEGO bricks renders

All renders were generated based on 3D models from LDraw library^7^ using Blender tool^13^ and its extension called ImportLDraw^14^. The renders simulate bricks moving on a conveyor belt as seen by a camera facing the belt. As a base, a default Blender scene was used. As a background, a semi-white plane was used. The lighting is composed of 2 area lights. The first one generates white light of power equal to 100 W and covers a square area of 3.07 m in size. The second one generates white light (H: 0.0, S: 0.0, V: 1.0) of power equal to 400 W and covers a square area of 3.39 m in size. The camera is of perspective type with a focal length of 10 mm. No scale was applied. For detailed parameters of each object please consult the scene file located in LegoSorter GitHub repository^15^ in dataset/scenes/simple.blend path.

For each render the brick is placed at the location X: 1.1479 m, Y: 0.0 m, Z: 4.0 m and is allowed to fall to the plain surface. The brick rotation was selected at random thus simulating the brick falling on the conveyor belt and thanks to using Blender physics and animation engine eliminated impossible positions, e.g. lying on a thin edge diagonally. The colors for the rendered LEGO bricks were selected from the list reflecting the most used colors of LEGO bricks (Table 1).

**Table 1:** The color codes used for rendering, that are representing the most common colors of LEGO bricks.

Color name	Hex code
White	0xFFFFFF
Brick Yellow	0xD9BB7B
Nougat	0xD67240
Bright Red	0xFF0000
Bright Blue	0x0000FF
Bright Yellow	0xFFFF00
Black	0x000000
Dark Green	0x009900
Bright Green	0x00CC00
Dark Orange	0xA83D15
Medium Blue	0x478CC6
Bright Orange	0xFF6600
Bright Bluish Green	0x059D9E
Bright Yellowish-Green	0x95B90B
Bright Reddish Violet	0x990066
Sand Blue	0x5E748C
Sand Yellow	0x8D7452
Earth Blue	0x002541
Earth Green	0x003300
Sand Green	0x5F8265
Dark Red	0x80081B
Flame Yellowish Orange	0xF49B00
Reddish Brown	0x5B1C0C
Medium Stone Grey	0x9C9291
Dark Stone Grey	0x4C5156
Light Stone Grey	0xE4E4DA
Light Royal Blue	0x87C0EA
Bright Purple	0xDE378B
Light Purple	0xEE9DC3
Cool Yellow	0xFFFF99
Medium Lilac	0x2C1577
Light Nougat	0xF5C189
Dark Brown	0x300F06
Medium Nougat	0xAA7D55
Dark Azur	0x469BC3
Medium Azur	0x68B3E2
Aqua	0xD3F2EA
Medium Lavender	0xA06EB9
Lavender	0xCDA4DE
White Glow	0xF5F3D7
Spring Yellowish Green	0xE2F99A
Olive Green	0x77774E
Medium-Yellowish Green	0x96B93B

During rendering each brick object was placed in 9 different positions separated by −1.5 m in the Y-axis direction (moving the brick down the conveyor belt). At each position, 10 images were created by randomly rotating the brick object on the Z-axis and/or flipping it upside down. Each time, to simulate different lighting conditions, a random selection of the available light sources was used (either the first, the second, or both aforementioned light sources were enabled). Afterward, empty, with no brick visible, and repeating frames, were removed from the set, thus in some cases, especially for larger bricks, a smaller number of images was generated. The images were saved in JPEG format in different resolutions to further simulate different quality of images. The bricks were then extracted from the original images using OpenCV^16^ edge detection algorithms.

### LEGO bricks photos

The photos available in *Tagged images with LEGO bricks* dataset^9^ contain bricks in various environmental surroundings found in a typical household, e.g. a box, on a carpet, on a keyboard, etc. and were taken using different cameras using their default settings to ensure diversity of the images. At first, randomly selected bricks were photographed and manually tagged by the dataset author. Each photo contains from 1 to 32 bricks in one photo. This set, combined with the renders (as described in the Data records section, *Tagged images with LEGO bricks* dataset^9^), was used to train a YOLO version 5^17^ neural network in its small variant^18^. The model, combined with our custom mobile app (Lego Sorter App^19^) and a python server application (Lego Sorter Server^20^) allowed quick creation of the 2nd part of the dataset. The model is publicly available as part of the Lego Sorter Server^20^ application.

**Fig. 2:**
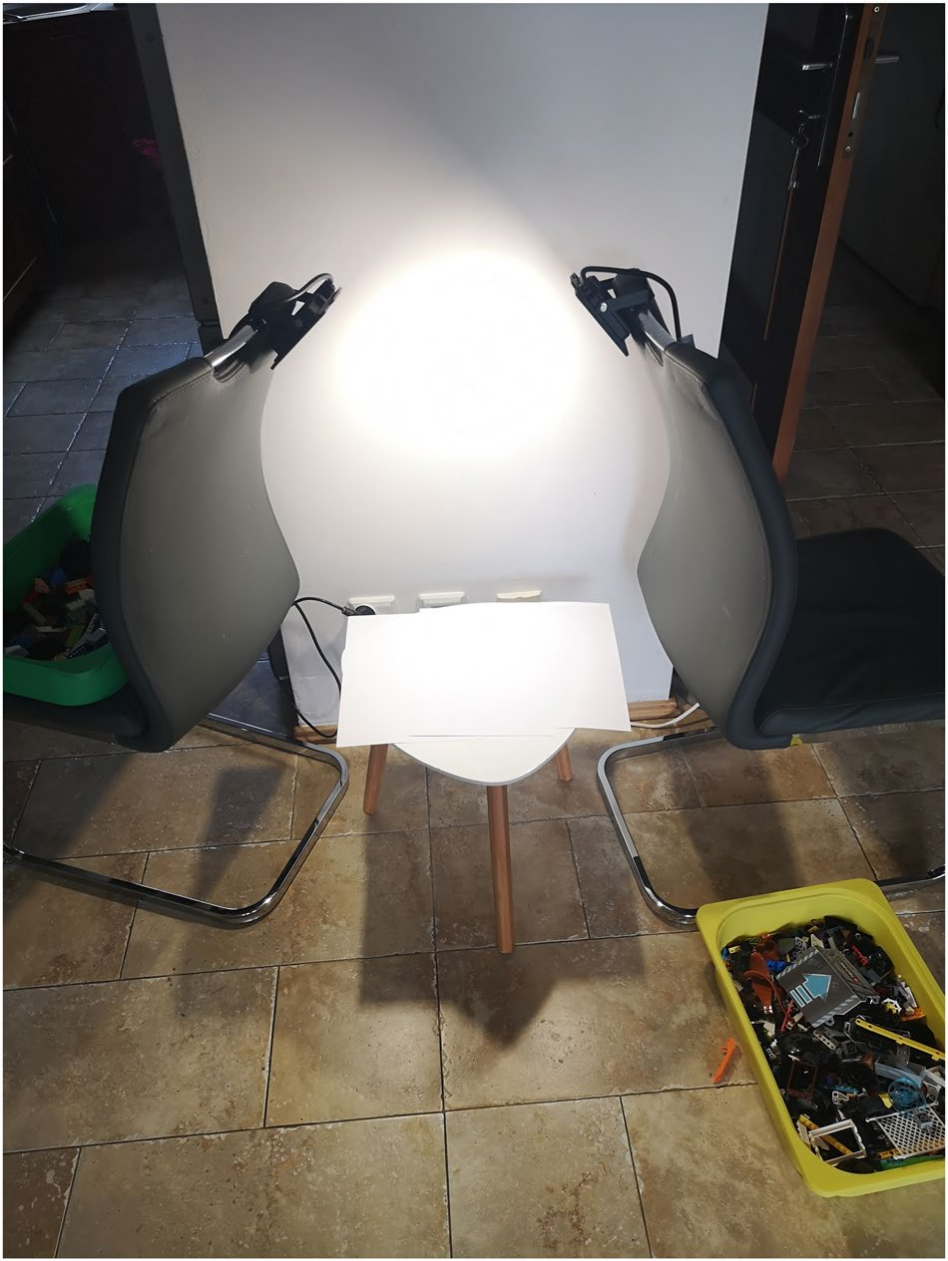
Photo stand for taking images of LEGO bricks. Bricks of the same shape were spread on the white surface and the photos were taken using a hoovering handheld camera. The bricks’ coordinates were automatically detected using a neural network.

In all other cases where photos were taken, the bricks were placed on the white, non-reflecting background, illuminated using two top-down facing 1600lm, 4000 K LED lamps, and photographed using a Huawei P20 Pro camera. The shutter speed and ISO were set to 1/100 and 50 respectively (to match the frequency of the LED lamps). All other settings were left at default values. The bricks photos were taken from random viewing angles ranging from 0° to 180° in relation to the photo base surface in all directions by a handheld camera hovering over the setup at the height of approximately 10–30 cm (Fig. 2). This allows the simulation of different viewing positions available for given brick types. The uniform background is the most versatile in AI-based sorting solutions. It also allows easy, automated background replacement by any texture.

The use of the aforementioned mobile app required the presorting of LEGO bricks which was done manually. During each session, a single class of bricks was automatically photographed while the user hovered the phone with the mobile app above the aforementioned photo setup with bricks scattered over a white desk covered with white, matte paper to reduce glare. The images were sent to a server where the brick locations in the images were detected. The server was responsible for the creation of bounding boxes according to the output from the YOLOv5 network. The images were then checked manually for errors and all partial images or wrongly detected objects were removed from the dataset. To simplify further processing the individual bricks were extracted from photos taken using the OpenCV library using bounding boxes defined by automatically detected coordinates.

Part 2 of the dataset^10^, thus contained only photos with bricks of one shape (but different, randomly selected colors and alignment), that were manually sorted out of the big pile of mixed bricks. This approach allowed quicker annotation of photos as the whole set from one photo session could be annotated at once.

In all cases, the brick coordinates were stored in XML files named identically as the image in PASCAL VOC format. Each photo can contain any number of bricks.

### Image resolution

All datasets contain images with varying sizes as denoted in the Data records section. This was done on purpose to ensure the diversity of the quality of the images. The extracted images done either by edge detection algorithms or based on neural network-generated bounding boxes will have sizes dependent on the shape and size of the extracted brick. In many cases even for the same shape, the final image size will be different depending on how the brick is placed, the viewing angle, etc. (e.g. brick number 10288).

### Extracted images similarity

Some LEGO bricks have very simple shapes (e.g. 22484, 2654, 3960, etc.) thus depending on the brick layout and camera viewing angle different photos might appear similar or even identical. We decided to not delete even very similar images as neural networks for the training process usually require as much data as possible and there might be important light or alignment changes between potentially similar images that can impact the training process.

### Image annotation

The images in *Tagged images with LEGO bricks* dataset^12^ were manually annotated using the labelImg tool. It is publicly available on the projects code repository at https://github.com/tzutalin/labelImg^21^.

## Data Records

**Fig. 3:**
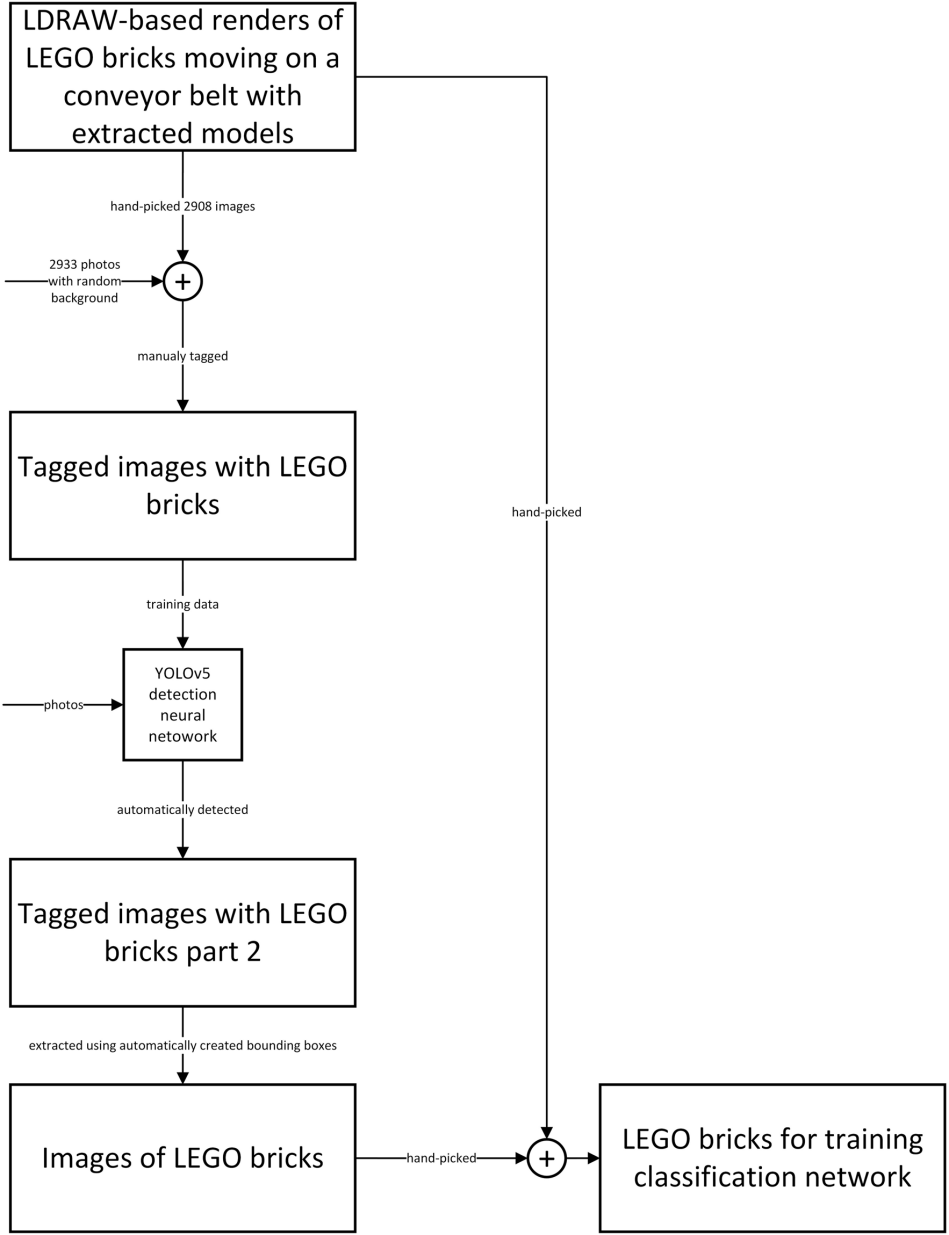
Relations between datasets.

The five datasets described in this paper are hosted using https://mostwiedzy.pl institutional repository and can be publicly accessed by their corresponding DOI identifiers. In all cases, all files are compressed into a single zip file. The total number of images in each dataset is shown in Table 2. If not stated otherwise all images were stored in JPEG file format and the binding boxes defining bricks coordinates are stored in XML files named as the associated image file in PASCAL VOC format^22^. The relation between the datasets is presented in Fig. 3.

**Table 2:** Number of real photos and renders in each dataset.

Dataset	Photos	Renders
*LDRAW-based renders of LEGO bricks moving on a conveyor belt with extracted models*^8^	0	935,967
*Tagged images with LEGO bricks*^9^	2,933	2,908
*Tagged images with LEGO bricks part 2*^10^	15,608	0
*Images of LEGO bricks*^11^	77,535	0
*LEGO bricks for training classification network*^12^	52,597	567,481

*LDRAW-based renders of LEGO bricks moving on a conveyor belt with extracted models* dataset^8^ (10.34808/xfgk-6f77) contains the renders created as described in the LEGO bricks renders section. The *original* folder contains the renders themselves and the *cropped_opencv* directory contains only bricks extracted from the renders using OpenCV^16^ edge detection algorithms. In both cases, the images were placed in a folder named after the LEGO brick code taken from the official LEGO catalog. The images have varying resolution ranging from 400 × 900 up to 1080 × 1920. The file naming convention is *brickID_colour_sequenceNumber_timestamp.jpeg*, where brickID is the official LEGO brick id number, color is the name of the selected brick color, sequenceNumber is the integer from 0 to 8 indicating the number of the image in the sequence simulating conveyor belt move and timestamp is UNIX time representation in milliseconds of the image creation time.

*Tagged images with LEGO bricks* dataset^9^ (10.34808/anq4-rn44) contains two types of images:

- 2933 photos containing from 1 to 32 LEGO bricks. The photos have varying resolution ranging from 228 × 220 up to 6000 × 8000. They contain random background and lightning conditions and present LEGO bricks from different angles.
- 2908 renders of LEGO bricks, 1 brick on each image handpicked from *LDRAW-based renders of LEGO bricks moving on a conveyor belt with extracted models* dataset^8^. The renders contain bricks in multiple shapes and colors, on a white background, taken from the camera facing bottom-up. The renders have varying resolution ranging from 400 × 400 up to 800 × 1200.

The images are named randomly and were placed in a respective subdirectory (photos and renders) and further in a subdirectory denoting the number of bricks visible on the image. The images were manually tagged with the coordinates of LEGO bricks bounding boxes created using labelImg software^21^. This dataset was used to train the YOLOv5 deep neural network for detecting LEGO bricks on images.

*Tagged images with LEGO bricks part 2* dataset^10^ (10.34808/7kk9-tn08) contains only photos of LEGO bricks. For each brick shape the bricks in the images have been randomly selected from the author’s collection so the brick’s colors couldn’t be previously planned and are thus chosen randomly as the dataset is oriented towards the discrepancy of LEGO brick shapes rather than colors. The images have varying resolution (1080 × 1920, 1539 × 2736, or 2160 × 3840). The images were tagged with the coordinates of LEGO bricks bounding boxes. The coordinates were generated automatically using a YOLOv5 neural network trained with the *Tagged images with LEGO bricks dataset*^9^ *as described in LEGO bricks photos section. The images are tagged by directory placement. The top-level directory is named after categories as found on the Rebrickable website*^6^ *and contains sub-directories named after the official LEGO brick number. If given LEGO brick is available under multiple brick numbers (e.g. 3004 and 3065) due to a slight construction change they are located in a single folder with brick number connected with an underscore (e.g. Bricks/3004_3065). Each image can contain any number of LEGO bricks (even 0) and only complete bricks are labeled. The dataset serves as an intermediate for the creation of Images of LEGO bricks*^11^ *and as such was not cleaned up after the automatic processing*.

*Images of LEGO bricks*^11^ (10.34808/arsb-4268) contain extracted images of LEGO bricks taken from automatically annotated data available in *Tagged images with LEGO bricks part 2* dataset^10^. Each image contains a single brick extracted using OpenCV^16^ library according to bounding boxes generated by the YOLOv5 network (as described in LEGO bricks photos section). Incorrectly extracted images, mainly due to occasional errors generated by the YOLOv5 network (e.g. only parts of bricks were visible) and photos containing other objects than a single LEGO brick were manually removed from the dataset. For each brick shape the bricks in the images have been randomly selected from the author’s personal collection so the brick’s colors couldn’t be previously planned and are thus chosen randomly as the dataset is oriented towards the discrepancy of LEGO bricks shapes rather than colors. The images are tagged by directory placement. The top-level directory is named after categories as found on the Rebrickable website^6^ and contains sub-directories named after the official LEGO brick number. If given LEGO brick is available under multiple brick numbers (e.g. 3004 and 3065) due to a slight construction change they are located in a single folder with brick number connected with an underscore (e.g. Bricks/3004_3065).

*LEGO bricks for training classification network*^12^ (10.34808/rcza-jy08) contains both renders and photos of LEGO bricks organized into 431 classes. Both the renders and images are hand-picked from *LDRAW-based renders of LEGO bricks moving on a conveyor belt with extracted models*^8^ and *Images of LEGO bricks*^11^ datasets. The images are tagged by directory placement. The two top-level directories (photos and renders) are named after their contents and contain sub-directories named after the official LEGO brick number where in turn images of bricks are located accordingly.

## Technical Validation

The *Images of LEGO bricks*^11^ datasets containing extracted photos were visually verified for wrongly and partially detected objects. In all cases, such images were removed from the dataset. The *Tagged images with LEGO bricks part 2* dataset^10^ was checked at random for correctness of bounding box placement and general quality of the detection performed by the YOLOv5 model. Identified tags of not complete bricks were removed from the XML files, however, the empty photos contained more bricks than found by the network or contained parts of the bricks were not removed, as the main purpose of this dataset was to create a high volume of extracted LEGO images.

**Fig. 4:**
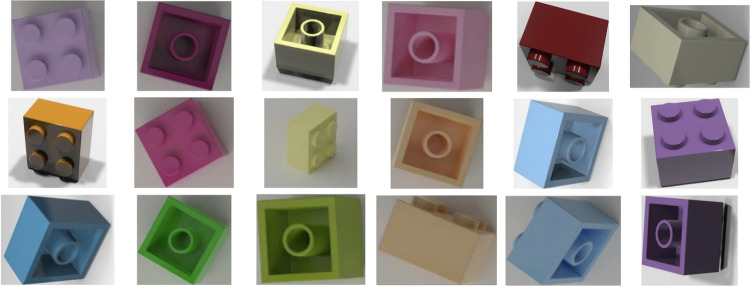
Sample renders for brick number 3003.

**Fig. 5:**
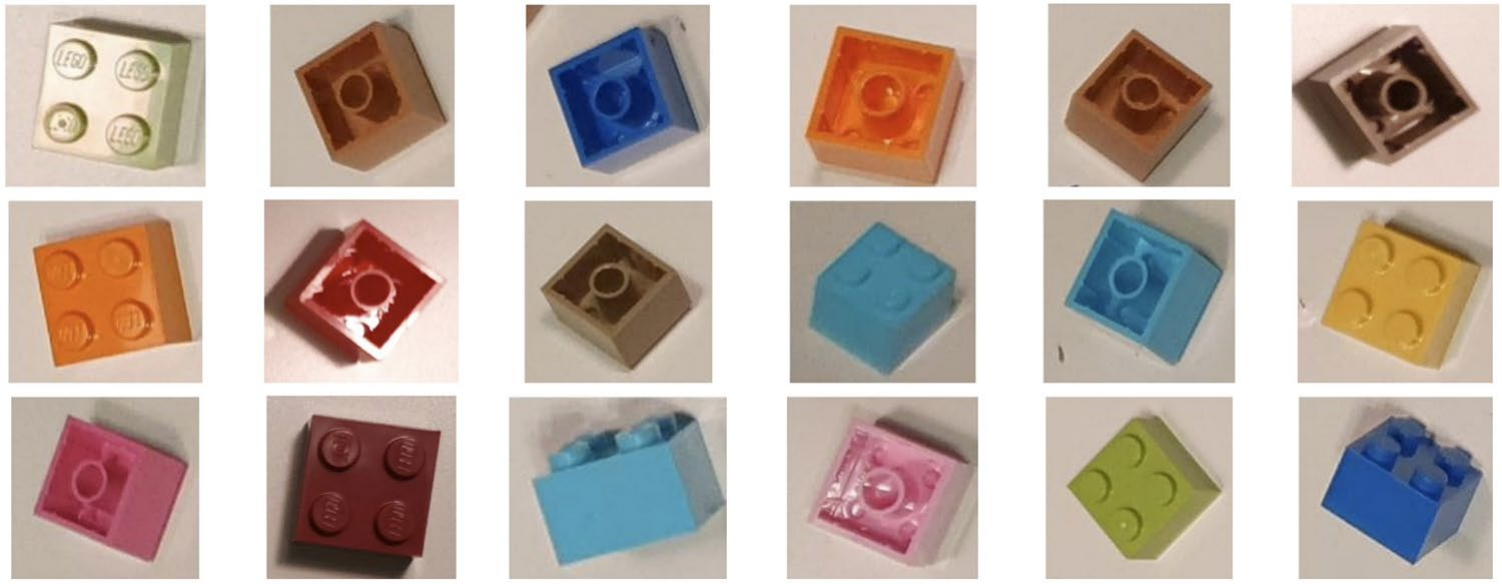
Sample real photos of brick number 3003.

In all cases, renders were verified automatically for the occurrence of empty images. For that purpose, the image was compared with a purposely made empty frame. All images were compared with the empty frame using the Structural Similarity Index algorithm^23^. When the score was 0.99 and higher the image was classified as an empty image. The *LDRAW-based renders of LEGO bricks moving on a conveyor belt with extracted models* dataset^8^ was afterward manually browsed to eliminate remaining empty renders. The quality of the renders was also visually checked by comparing them with real photos of LEGO bricks. Sample renders, after being cropped, can be seen in Fig. 4 whereas sample real photos of the same brick ID can be seen in Fig. 5.

The quality of renders was also checked using deep models for object detection and classification. For direct verification *Tagged images with LEGO bricks*^9^ and *LEGO bricks for training classification network*^12^ datasets (as the second dataset is composed of images from remaining three datasets).

**Fig. 6:**
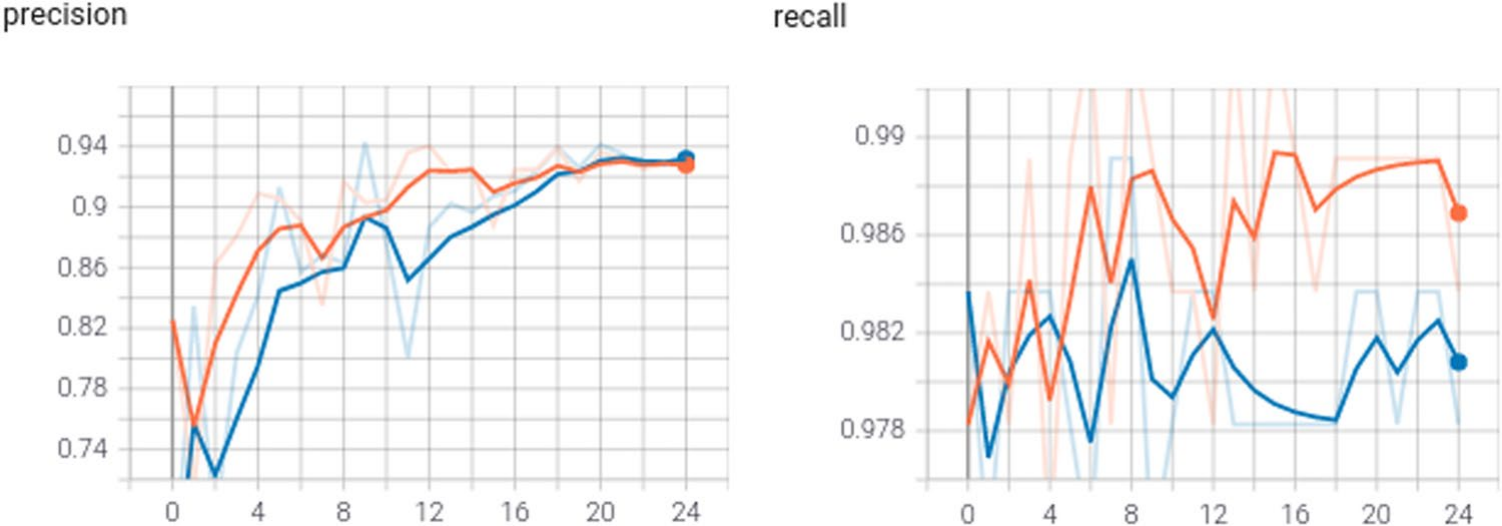
Precision and recall comparison of YOLOv5 small (blue) and medium (red) models trained using *LDRAW-based renders of LEGO bricks moving on a conveyor belt with extracted models* dataset^8^.

**Fig. 7:**
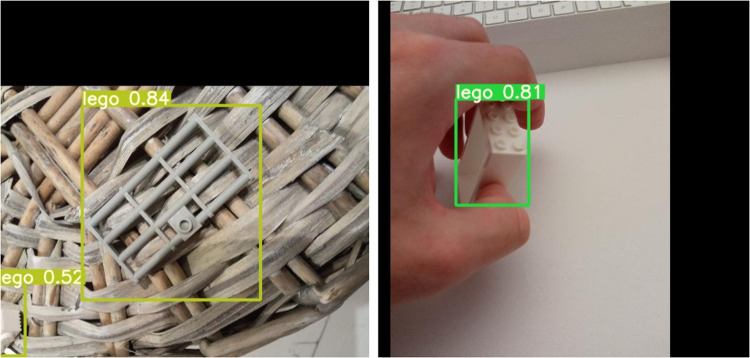
Sample LEGO brick detection results done by YOLOv5 small model trained using *LDRAW-based renders of LEGO bricks moving on a conveyor belt with extracted models* dataset^8^.

For object detection, we used the *Tagged images with LEGO bricks*^9^ dataset^24^ by training small and medium YOLO (You Only Look Once) version 5 models (YOLOv5s and YOLOv5m respectively^17,18^). The models were used in their default settings and transfer learning was applied. The dataset used to train the networks contained a mixture of renders and real photos and the test subset contained real photos only. The test set contained 880 images (around 30%) randomly selected from the real photos in the dataset. The training set was composed of the remaining photos and the renders. The process proved to work very well, both models achieved good values of precision and recall (Fig. 6). Despite the datasets used composed of renders and photos of bricks on a white background, as can be seen in Fig. 7 the network was able to detect never seen before LEGO bricks even in complicated scenarios proving the generality of the datasets.

To test *LEGO bricks for training classification network*^12^ dataset we trained EfficientNetB0^25^ and ResNet50^26^ classification networks. The training set is composed of randomly selected 447000 images - 650 renders and 350 photos for each of the 447 classes available in the dataset. The test set was also randomly selected and composed of 50 renders and 50 real photos. EfficientNetB0 network was trained using both the whole training set and only the 650 renders selected for each class and ResNet50 was trained using only the whole training set (renders and real photos). Similarly, as in the previous test, the models used default parameters, and transfer learning was applied. The full training procedure can be found in^27^.

**Fig. 8:**
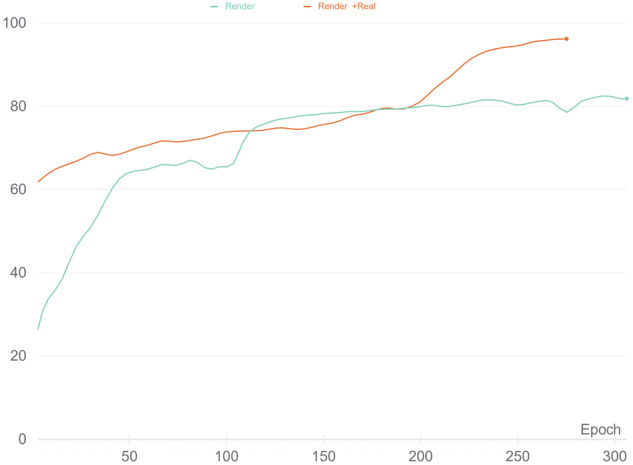
Precision of bricks labeling for EfficientNetB0 network trained using *LEGO bricks for training classification network*^12^ using renders only (blue) and renders with real photos (red).

EfficientNetB0 model achieved very good results, with efficiency reaching 80% when only renders were used for training and almost 100% when both renders and photos were used (Fig. 8). The ResNet50 model achieved 93.81% of Top1 accuracy and 99.10% of Top5 accuracy.

**Fig. 9:**
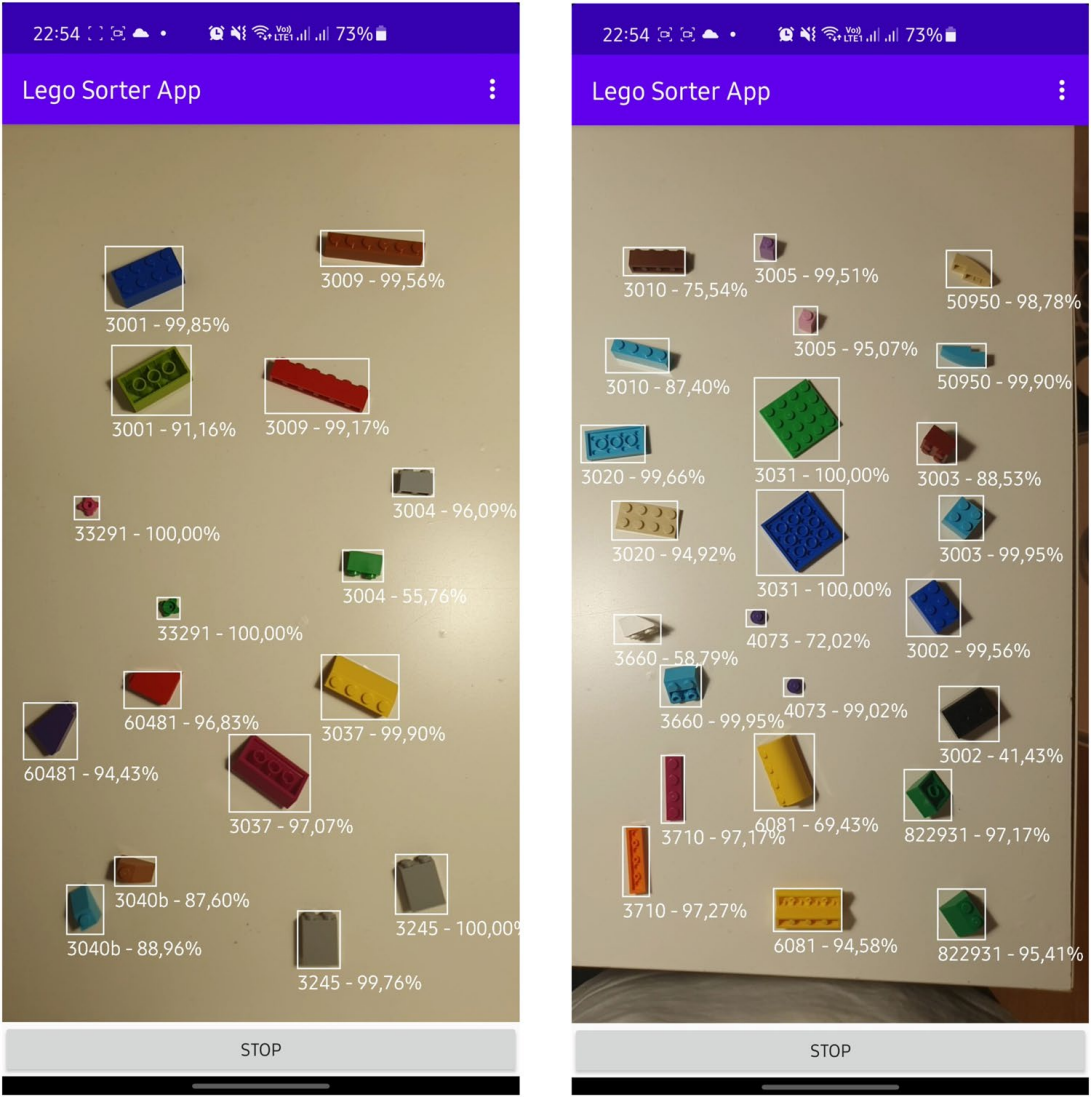
Real-time tests for ResNet50 model trained using *LEGO bricks for training classification network*^12^.

**Fig. 10:**
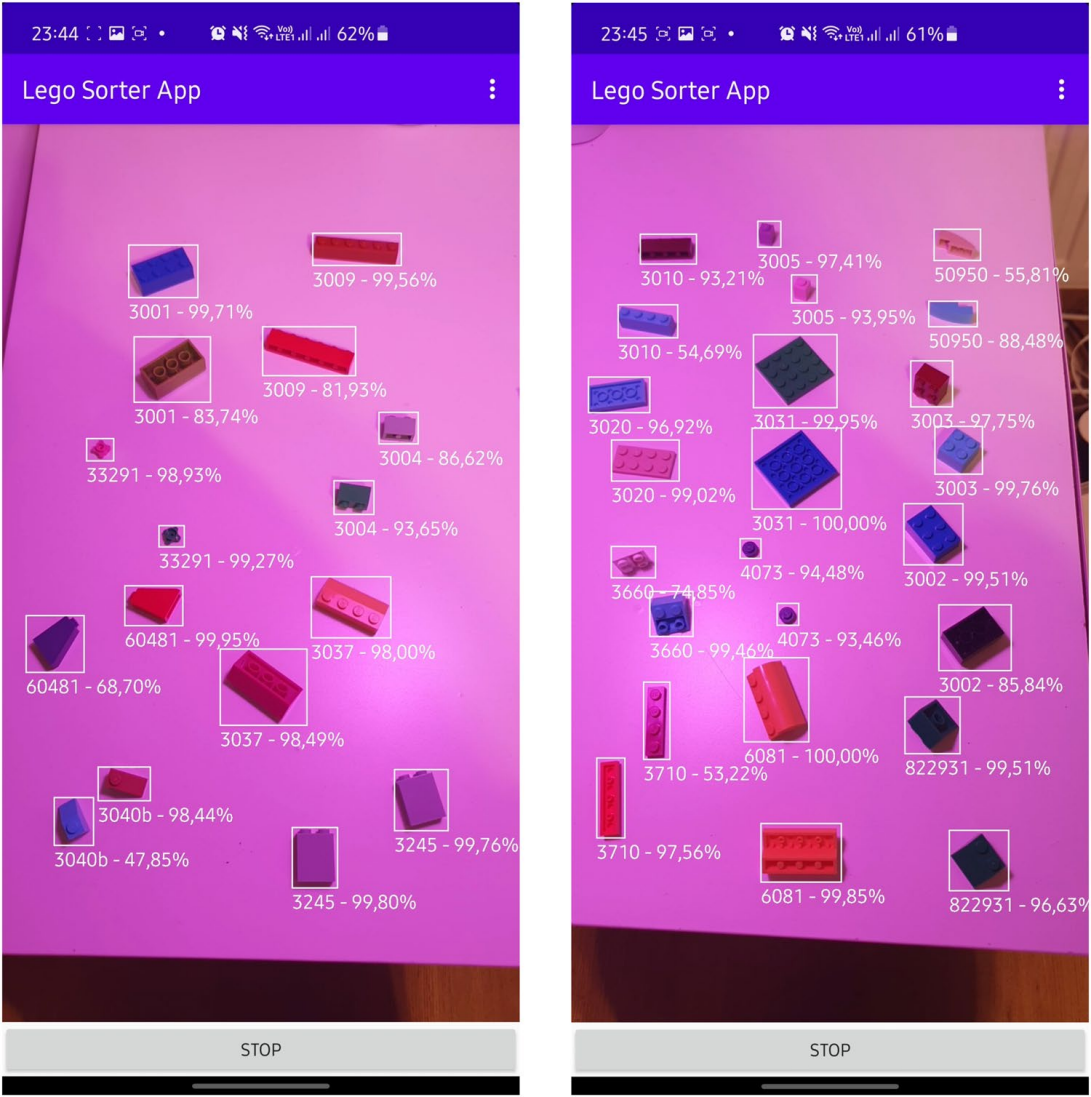
Real-time tests for ResNet50 model trained using *LEGO bricks for training classification network*^12^–pink light.

We also combined the aforementioned and trained YOLOv5s and ResNet50 models within a Lego Sorter App^19^ and used it to detect and classify LEGO bricks in never used before setup where photos were taken in real-time. In this scenario we were able to detect and classify bricks both on a white background and in significantly changed lightning conditions with 100% accuracy (e.g. as seen in Figs. 9, 10).

## Data Availability

Custom tools used to take photos, generate renders, annotate photos, and extract annotated bricks from the complete scene, including the trained neural networks, are publicly available through the Lego Sorter project^15^ and its repositories available at https://github.com/LegoSorter.
